# Anesthesia Related Toxic Effects on In Vitro Fertilization Outcome: Burden of Proof

**DOI:** 10.1155/2015/475362

**Published:** 2015-06-16

**Authors:** Paraskevi Matsota, Eva Kaminioti, Georgia Kostopanagiotou

**Affiliations:** 2nd Department of Anesthesiology, School of Medicine, University of Athens, “Attikon” Hospital, Rimini 1 Street, Chaidari, 12462 Athens, Greece

## Abstract

Management of pain and anxiety during oocyte retrieval makes anesthesia an important part of the in vitro fertilization (IVF) procedures. There are many studies investigating the influence of anesthesia on IVF success. This review article provides an overview of published data regarding the potential toxic effects of different anesthetic techniques (Loco-regional, general anesthesia (GA), and monitored anesthesia care (MAC)), different anesthetic agents, and alternative medicine approach (principally acupuncture) on the IVF outcome. From our analysis, evidence of serious toxicity in humans is not well established. Trials regarding different anesthetic techniques ended up without clear conclusions. Studies about GA came up with conflicting results. A few trials relate GA with lower pregnancy rates, although some others failed to prove this conclusion. Furthermore, detectable amounts of some anesthetic agents are measurable in the follicular fluid but these findings are not strongly associated with toxicity. MAC and Loco-regional anesthesia appear as safe alternative choices and there is evidence of improved outcome. Whereas acupuncture may provide assistance increasing IVF success according to some trials, some others could not obtain these effects. Questions about the appropriate time of application and the underlying mechanism of action are not answered yet, so further investigation should be done.

## 1. Introduction

Assisted reproductive techniques (ART) are methods used to achieve pregnancy by artificial or partially artificial means. Last decades ART are widely used primarily for infertility treatments.

In vitro fertilization (IVF) is the technique of letting fertilization of the male and female gametes (sperm and egg) occur outside the female body and it is the most prevalent technique of ART. It involves the five following steps.

**Step 1 (ovarian stimulation).**

Women are given medicines (fertility drugs) in order to make the ovaries produce several eggs (to boost egg production).

**Step 2 (egg collection (follicular aspiration)).**

The aim of this minor surgery is to remove the eggs from the woman's body, guided by ultrasound images (transvaginal oocyte retrieval). The operator inserts a thin needle through the vagina into the ovary and sacs (follicles) containing the eggs. The needle is connected to a device which sucks the eggs out of each follicle, one at a time.

**Step 3 (fertilization).**

The best quality eggs and sperm are stored in an environmentally controlled chamber. The sperm enters an egg and this procedure is called fertilization. If the chance of fertilization is low, the sperm may be directly injected into the egg. This is called intracytoplasmic sperm injection (ICSI).

**Step 4 (embryo culture).**

The fertilized egg divides and becomes an embryo.

**Step 5 (embryo transfer back into the uterus of the female).**

Embryos are placed into the woman's womb, while the woman is awake, 3–5 days after follicular aspiration. The doctor inserts a catheter containing the embryos into the woman's vagina up into the womb. Pregnancy results when an embryo implants in the lining of the womb and grows.

Anesthesiologists are mainly involved at the second step of the above described procedures. Women undergoing transvaginal oocyte retrieval are anxious and experience mild to moderate pain caused by the puncture of the vaginal skin and ovarian capsule with a needle in order to aspirate the oocytes. Repeated attempts are often necessary before success is achieved. Thus, it becomes important to ensure a comfortable environment for them minimizing their pain and anxiety and improving their cooperation. For these reasons, sedation alone or combined with analgesia, as well as different anesthetic techniques including general anesthesia, regional anesthesia, and alternative medicine approach, has been used during these procedures. All the above techniques demand the assistance of an anesthesiologist in order to make transvaginal oocyte retrieval a safe and effective procedure for women and clinicians.

Many studies have been performed investigating the effect of anesthesia on the IVF outcome but have yielded conflicting results. These controversies are mainly due to the fact that different methodologies have been used including different anesthetic techniques, different anesthetic agents, and different outcome parameters under investigation such as number of collected and matured oocytes; embryo quality fertilization; cleavage; implantation; abortion; pregnancy and delivery rates; plasma and follicular fluid concentrations of the agent; plasma and follicular levels of prolactin, progesterone, and cortisol; and plasma levels of immunoreactive beta-endorphin.

The present review is aimed at investigating the existing literature (up to 2014) regarding the effect of traditional anesthesia and alternative medicine approach on IVF outcome. The primary outcome measure is the toxic effects on one or more of the following IVF outcome parameters: pregnancy rates, fertilization rates, and number and quality of oocytes retrieved.

## 2. Materials and Methods

### 2.1. Trial Identification

Published trials, written in English and investigating the effect of anesthetic agents, anesthetic techniques, and alternative medicine used for the assisted reproductive technologies, on their outcomes were identified and sought by searching on Pubmed and ISI Expanded, up to 2014, with no time limitation. They were searched using the following headings, key words: anesthesia, IVF outcome, oocytes aspiration, fertilization, analgesia, anesthetics, pregnancy rate, acupuncture, and reproductive technology.

### 2.2. Inclusion Criteria

This review was limited to prospective trials (most of them were randomized) and retrospective studies, written in English and investigating the anesthetic and alternative medicine impact on IVF outcome. Specifically, we included studies using either traditional anesthetic procedures or alternative medicine (acupuncture) for pain relief during ART and only trials that assessed IVF outcomes in terms of different parameters such us pregnancy rate, fertilization rate, number and quality of oocytes collected, embryo quality, and follicular levels of an anesthetic agent possibly related to toxicity. All these studies were conducted after consent had been obtained by all participants, while their results were referred to only in women and not in IVF cycles. The unit of analysis was per participant and not per cycle. Multiple live births were counted as one live birth event.

### 2.3. Exclusion Criteria

Participants in the trials had to meet all the above criteria to be included in the review. We excluded studies with evidence of inadequate sequence generation (such as small patients number) as they are associated with a high risk of bias. Trials in animals were excluded, too.

## 3. Results and Discussion

*Trials Included.* Of the 115 trials identified, only 43 met the selection criteria for this review and were included in the analysis; 42 studies appeared to be reviews, meta-analyses, pilot studies, or case reports; 7 studies were excluded because they measured different parameters than those related to IVF outcome (pain and nausea) or their conclusions were referred to in cycles and not women; 7 trials were in animals; 11 studies were not appropriate as they were written in different languages (10 were written in Chinese and 1 was written in Spanish); 2 trials were not included as they are ongoing and 3 studies were referred to in herbals. All prospective trials are randomized, except 5 prospective controlled trials, investigating the follicular fluid concentration of some agents.

**Figure 1:**
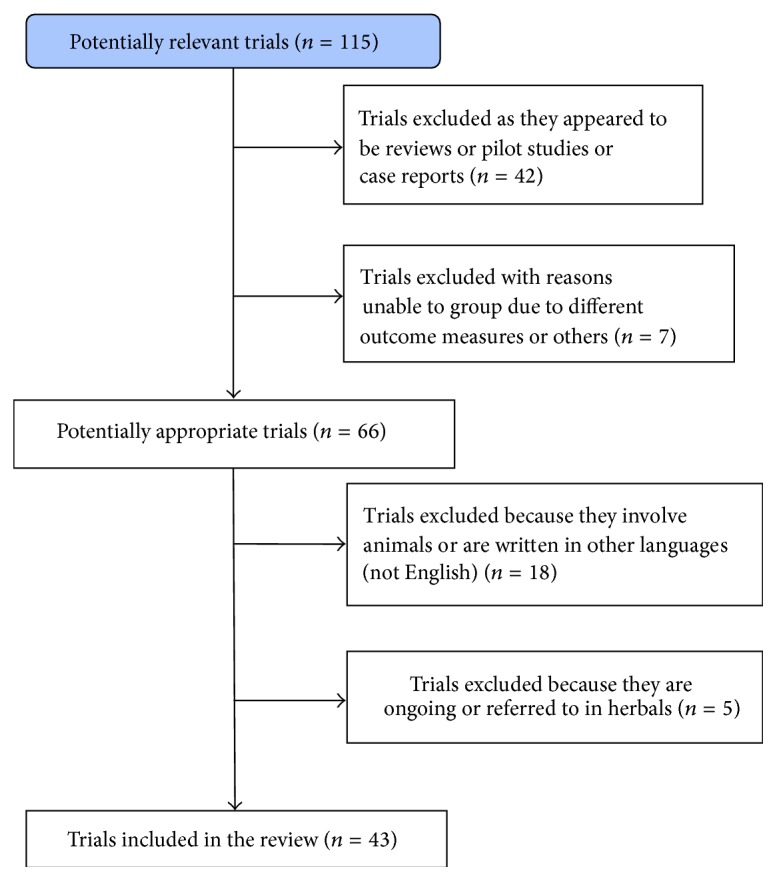
The trial flow.

The trial flow is illustrated in Figure 1.

*(I) Traditional Anesthetic Techniques and Agents. *Consider the following.

*(1) Anesthetic Techniques and IVF Outcome.* Infertility is a source of anxiety for women. Furthermore, pain during oocyte retrieval makes IVF procedures very uncomfortable for them. These characteristics prove the fundamental role of an anesthesiologist during ART. The anesthetic techniques that can be used for IVF are

1. Loco-regional anesthesia (spinal, epidural, and blocks),
2. general anesthesia (GA),
3. MAC, monitored anesthesia care, and sedation.

There are 9 comparative studies investigating the effect of these techniques on IVF outcome that met the selection criteria (Table 1).

Recent trials concluded that the use of spinal anesthesia improves IVF outcome [1, 2]. Spinal anesthesia increased significantly the chance of IVF success in a randomized prospective trial by Azmude et al. in 2013 [1]. According to a cohort study based on analysis of patient records at Mirza Kochak Khan Hospital, Tehran University of Medical Sciences (Aghaamoo et al., 2014), it was recommended to use spinal anesthesia instead of GA for oocyte retrieval, to achieve successful in vitro fertilization outcome [2]. Nerve blocks (ovarian block and paracervical block) as an additional method for pain relief proved no harm on the IVF success [3, 4]. Epidural anesthesia is not commonly used but it seems a safe alternative choice, as it was not associated with toxic effects [5].

General anesthesia can be performed with intravenous or inhalation agents, and coadministration of sedatives and analgesic drugs. Studies on the potential toxicity of general anesthesia ended up with conflicting results.  Table 2 summarizes the details of the 5 studies about GA's potential toxicity. GA compared to MAC revealed statistically significant difference only with regard to pregnancy rate; the prevalence of MAC appeared clear enough [6]. On the contrary, two other studies failed to prove this conclusion. There were no significant differences in pregnancy rates between general anesthesia and sedation or paracervical local anesthetic block [7, 8].

MAC is well tolerated and easy to deliver in day case procedures, such as oocytes aspiration. MAC with remifentanil had a greater pregnancy rate than GA in a retrospective data analysis conducted by Wilhelm et al. [6] in 2002, investigating 251 women undergoing IVF. In 2008, Milanini et al. [9] showed that sedation with intravenous infusion of remifentanil does not interfere in the quality of oocytes retrieved and embryo score. MAC with remifentanil seems to be a safe anesthetic option and it is related to an improved IVF outcome. 

*(2) Anesthetic Agents and IVF Outcome.* In vitro fertilization is a procedure with short duration. Ideal anesthetic agents should have rapid onset of action and rapid recovery time. They should not accumulate in the follicular fluid and they must not have toxic effects on the outcome. 

*Benzodiazepines.* Benzodiazepines are used for premedication, procedural sedation, and supplementation of general or regional anesthesia. A common sequel to intravenous administration of benzodiazepines is anxiolysis and anterograde amnesia. These two main characteristics of these drugs make them suitable for patients undergoing unpleasant or repeated procedures, like oocytes retrieval. Midazolam is the most commonly used benzodiazepine during oocyte retrieval and diazepam has its role as an agent of premedication. Unfortunately, bibliography about toxic effects of benzodiazepines on IVF outcome is poor and no study met the selection criteria of this review. 

*Intravenous Anesthetic Agents.* Propofol is the most commonly used intravenous anesthetic agent in sedation and general anesthesia. Its pharmacokinetic profile makes propofol anesthetists' first choice. Several studies investigate the effect of this agent on IVF success with conflicting results.

Of the studies investigating toxicity, two of them relate propofol with negative effects on the reproductive outcome [10, 11] and five studies conclude with the opposite result [12–16]. According to these findings propofol is probably a safe choice, but cautious use is recommended. Propofol also accumulates in the follicular fluid [15, 17].

Thiopental proved to be a safe alternative choice to propofol, as it was not associated with toxic effects [13, 14].

There were no studies investigating potential toxicity of the other hypnotics, like ketamine or etomidate, as part of the GA's protocol. 

*Opioids.* Women can be offered adequate pain relief. For this reason, opioids are used in oocyte retrieval procedure primarily for their analgesic effects. The most frequently used are fentanyl, alfentanil, and remifentanil, because of their pharmacokinetic profile that enhances fast track anesthesia. Pethidine is used in some cases as an agent of premedication.

The amount of alfentanil [18] or remifentanil [19] is not associated with adverse effects on fertilization rate, embryo development, or clinical pregnancy rate.

Remifentanil in clinical practice is superior to fentanyl [20]. The pregnancy rate was significantly higher after remifentanil than after fentanyl. This study suggested that the likelihood of a successful pregnancy was higher with a remifentanil-based MAC technique than with a fentanyl-based MAC technique. 

*Local Anesthetics.* Blocks are used in order to mediate pain during transvaginal oocyte retrieval. Paracervical block (PCB) proved to be very useful. The most commonly used local anesthetic agent is lidocaine. Fertilization, cleavage, and pregnancy rates did not differ significantly in women with and without PCB (with 50 mg lidocaine) [21]. IVF outcome was not affected even by bigger doses of lidocaine in a PCB (150 mg versus 200 mg) [22]. Therefore, the use of 50 mg of lidocaine in PCB is recommended because of the lack of improvement in pain relief on higher doses and potential dose-related risks [23]. There are limited studies investigating potential toxicity of the other local anesthetics and no one met the selection criteria. 

*Inhalation Agents.* Some studies in animals are conducted investigating toxic effects of inhalation agents (isoflurane and nitrous oxide) on IVF success [24, 25]. These studies indicate that embryo toxic effects can be detected and that these anesthetics may be detrimental to the success of in vitro fertilization and embryo transfer. These studies are excluded from this review, as they did not meet the selection criteria. The effect of nitrous oxide on human's in vitro fertilization success rate was poorly studied and the results did not clearly confirm the findings in animals [26]. 

*Follicular Fluid Concentrations of Anesthetic Agents.* The proportion of the anesthetic agent that reaches the follicular fluid (FF) can indirectly reflect the likelihood of the agent's toxicity on the IVF outcome. Only a small number of studies investigate this crucial parameter. Some of them use a small number of patients and that means that further investigation should be done in order to reach a safe conclusion.

Endler et al. [27] in 1987 measured the follicular fluid concentrations of thiopental and thiamylal during laparoscopy for oocyte retrieval. In both groups, measurable amounts of the respective drug were found in all FF aspirates.

Wikland et al. [21] in 1990 studied the concentration of lidocaine in follicular fluid and proved that there was no significant difference in lidocaine concentration between follicles containing oocytes that were fertilized and those that were not. Furthermore, the fertilization, cleavage, and pregnancy rates did not differ. According to them, it seems that the concentration of lidocaine found in the follicular fluid after PCB with 50 mg lidocaine does not negatively affect fertilization of the human oocyte or early cleavage of the human embryo.

Soussis et al. [28] in 1995 concluded that midazolam, fentanyl, and alfentanil were found in FF after a single intravenous dose, but there were no significant differences in fertilization or pregnancy rates in the three groups.

Propofol concentration in arterial blood and follicular fluid was investigated in a sample of 30 women by Christiaens et al. [17] in 1999. They found that there was no correlation between the concentration of propofol in the follicular fluid and the arterial blood concentration of the drug. They concluded that a propofol-based anesthetic technique resulted in significant concentrations of this agent in follicular fluid, related to the dose administered and to the duration of propofol administration. Follicular fluid concentrations of propofol were investigated in 11 cases of the 130 women participating in Ben-Shlomo et al. [15] study in 2000, as mentioned before. There was an increase of FF propofol concentration from the first to the last follicle (all had a higher concentration of propofol in the last FF as compared to the first (*P* < 0.01)), but no difference was found in the ratio of mature to immature oocytes. Nor were any differences found in fertilization, cleavage, and embryo cell number. 

*(3) IVF Outcome Parameters. *Consider the following.

*Pregnancy Rates.* Pregnancy is the main goal of the IVF procedure. Twenty-one studies were included in this review investigating IVF outcome using pregnancy rates, as the main parameter. Pregnancy rates were defined as follows:

- Chemical pregnancy rate defined as elevation of *β*-hCG more than 15 IU, 11 days after embryo transfer.
- Clinical pregnancy rate defined as evidence of a gestational sac with fetal heart motion measured at seven to eight weeks, confirmed with ultrasound.
- Ongoing pregnancy rate defined as evidence of a gestational sac with fetal heart motion at greater than twelve weeks (12 to 18 weeks), confirmed with ultrasound.
- Live birth rate defined as delivery of a live fetus after 20 completed weeks, gestational age.

Two studies comparing general anesthesia (GA) with spinal anesthesia (SA) proved that SA had an advantage on IVF success. Women undergoing SA had increased pregnancy rates compared with women who received GA for IVF [1, 2]. Epidural anesthesia and sedation (propofol and mask-assisted ventilation with nitrous oxide) had the same outcomes in terms of pregnancy [5]. No difference was found between performing preovarian block and paracervical local anesthetic block for oocyte retrieval [4].

Studies investigating GA versus MAC concluded with conflicting results. MAC technique with a remifentanil infusion was related with increased pregnancy rates compared to GA (with alfentanil, propofol, nitrous oxide, and isoflurane) [6]. On the other hand, some studies came with the opposite conclusion. Sedation with midazolam, diazepam, or propofol (according to clinical needs) did not prove superior to GA with a combination of remifentanil with either propofol or isoflurane in hypnotic concentrations [8]. GA with fentanyl, propofol, and isoflurane did not influence pregnancy rates compared with a sedative protocol consisting of midazolam and ketamine [12]. GA with propofol versus paracervical local anesthetic block for oocyte retrieval did not prove harmful on pregnancy rates [7]. Significant differences on pregnancy rates were not found between anesthesia (with propofol and alfentanil) and analgesia (with remifentanil) for transvaginal oocyte retrieval [16].

The percentage of pregnancies was greater in women receiving isoflurane/nitrous oxide compared with women taking propofol/nitrous oxide for ART [10]. Addition of nitrous oxide in women receiving isoflurane did not reduce in vitro fertilization pregnancy rates [26].

The comparison of different anesthetic methodologies for sedation during in vitro fertilization procedures (EMLA cream, propofol, thiopental sodium, and sevoflurane) showed no differences in pregnancy rates [11]. Comparing 2 different anesthetic agents, propofol and thiopental proved to be equally safe [13, 14].

Pregnancy rate was significantly higher after remifentanil infusion than fentanyl [20]. Different doses of alfentanil [18] and remifentanil [19] did not seem to influence pregnancy rates. Midazolam, fentanyl, alfentanil [28], and lidocaine [21] were detectable in the follicular fluid, but they did not affect pregnancy rates. Lidocaine and its dosage were not related to reduced pregnancy rates [22, 23]. 

*Fertilization Rate.* Fertilization is defined as the union of a human egg and sperm. The result of this union is the production of a fertilized egg, initiating prenatal development. Fertilization rate is defined as the proportion of oocytes resulting in two pronuclei formations.

Only one study concluded that fertilization rate was influenced by different anesthetic techniques for sedation during in vitro fertilization procedures. Women undergoing IVF and receiving EMLA or sevoflurane had similar fertilization rates but significantly higher than those who received propofol or thiopental sodium [11].

GA [7, 8, 12, 15], MAC, and epidural anesthesia [5] did not affect fertilization rates. Opioids did not seem to interfere with fertilization [16, 18–20, 28]. Propofol and thiopental [13, 14] proved to be equally safe. Nitrous oxide was not related to anomalous fertilization [26]. Local anesthetic blocks with lidocaine did not prove harmful [4, 21, 22]. 

*Number and Quality of Oocytes.* The number and quality of oocytes determine the possibility of a successful pregnancy. Of the 14 trials included in this review, investigating the number and quality of the oocytes, only two found significant differences: the number of collected oocytes was significantly higher with general anesthesia (remifentanil with either propofol or isoflurane) than with sedation (midazolam, diazepam, or propofol) [8]. Comparing EMLA cream versus propofol versus thiopental sodium versus sevoflurane, in the EMLA cream and thiopental sodium groups was observed the highest percentage of mature oocytes at metaphase II stage (MII). Anesthesia with sevoflurane had a lower percentage of good embryos [11].

Intravenous infusion of remifentanil does not interfere in the quality and number of oocytes retrieved compared with local anesthesia, but it makes easier the pick-up of oocytes because women do not experience pain during the procedure. In this way, it may be possible to recover more oocytes [9].

GA with propofol did not seem to interfere in oocytes retrieved compared with a paracervical local anesthetic block [7]. Hypnotic agents (propofol and thiopental) did not prove to be harmful, too [13–15]. Midazolam/ketamine sedative combination compared with fentanyl/propofol/isoflurane anesthesia for oocyte retrieval showed no differences in oocytes number and quality [12]. Studies on opioids, such as fentanyl, alfentanil, and remifentanil, showed no differences [16, 18, 20]. Lidocaine is a safe choice for a local anesthetic block, regardless of its dose [22, 23] and the type of block (preovarian block or paracervical block) [4]. 

*(II) Alternative Medicine Approach.* Alternative medicine is any practice that is put forward as having the healing effects of medicine but is not founded on evidence gathered using the scientific method. It consists of a wide range of health care practices, products, and therapies. Examples include new and traditional medicine practices such as various forms of acupuncture. Acupuncture is one of the most commonly used alternative medical procedures in the world.

Acupuncture is the stimulation of specific acupuncture points along the skin of the body that represent various organs and ailments, involving various methods such as penetration by thin needles or the application of heat, pressure, or laser light. Traditional acupuncture involves needle insertion, moxibustion, and cupping therapy. It is a form of alternative medicine and a key component of traditional Chinese medicine. Stimulating specific acupuncture points corrects imbalances in the flow of qi that are believed to cause diseases, through channels known as meridians.

The role of acupuncture in assisted reproductive techniques (ART) is under investigation for several years. For certain, it is one form of complementary and alternative medicine considered by women seeking assistance during transvaginal oocyte retrieval.

Paulus et al. [29] in 2002 showed that acupuncture seems to be a useful tool for improving pregnancy rate after ART. This trial was followed by a big number of studies, trying to further explore the role of individualized acupuncture treatment on IVF pregnancy rates. Parameters under investigation are the underlying mechanism [30], the magnitude of the time that acupuncture is performed, the ideal treatment frequency, and the placebo effect.

Despite some evidence suggesting beneficial effects of acupuncture on in vitro fertilization (IVF) success rates, some clinical trials could not duplicate these effects. Of total 16 trials for acupuncture that met the selection criteria of this review, 8 studies confirm the beneficial role of acupuncture on IVF outcome [29, 31–37], 6 studies failed to find significant differences [38–43], and 2 studies concluded that the use of acupuncture reduces IVF outcome [44, 45].

More research is needed with a greater number of subjects to elucidate the role of acupuncture on IVF outcome. Real and sham acupuncture should be investigated in more detail.

## 4. Conclusions

So far, literature regarding the impact of different anesthetic techniques and agents on IVF is not definite. Therefore, evidence based conclusions cannot be extracted. Findings about Loco-regional anesthesia probably appear more comforting. Spinal anesthesia and epidural anesthesia seem superior compared to GA. Nerve blocks (ovarian block and paracervical block) performed as the only anesthetic method or as an additional method for pain relief did not prove harmful. MAC seems a safe alternative choice. Specifically, MAC with remifentanil is related with increased reproductive outcome. Despite some evidence suggesting negative effects of GA on IVF success rates, some clinical trials failed to prove this conclusion.

Some anesthetic agents (propofol, thiopental, midazolam, fentanyl, and alfentanil) can accumulate in the follicular fluid. This can be used only as an indirect index of potential toxicity but cannot prove any real toxic effect on the outcome. The role of propofol appears controversial in some cases but generally is considered as a safe choice. Thiopental is not associated with toxic effects. There are no data on ketamine and etomidate.

The most commonly used opioids during IVF procedure are fentanyl, alfentanil, and remifentanil. Pethidine is only used as an agent of premedication. Research gives precedence to remifentanil, although fentanyl and alfentanil can be used as alternatives.

Acupuncture and its mechanism of action are not well known yet. The magnitude of the application time, the ideal treatment frequency, and the placebo effect are some of the issues seeking for answers. There are several studies investigating potential toxic effects on the IVF outcome but the conclusions are not clear enough.

In conclusion, studies in different anesthetic techniques and agents failed to give prominence to the ideal one. The best practice is cautious use of the anesthetic agents. Their potential toxicity is not well established in humans yet, although some trials present undesirable effects on the IVF outcome.

Literature about alternative medicine approach on the IVF outcome is very wide, but knowledge about acupuncture is not enough. Further investigation should be done to clarify the underlying mechanisms of action and the likelihood of negative effects or benefits on the reproductive outcome.

## Figures and Tables

**Table 1:** Details of trials comparing different anesthetic techniques for in vitro fertilization.

First author/year	Technique versus technique	Group size	Pregnancy rates	Fertilization rate	Cleavage	Oocytes (number and quality)
Aghaamoo et al. 2014 [2]	GA versus spinal analgesia	164 total Spinal group (*n* = 81) GA group (*n* = 83)	Spinal anesthesia is significantly related to increased chance of chemical pregnancy (*p* = 0.043)	—	—	—

Azmude et al. 2013 [1]	GA versus spinal anesthesia	200 total GA group (*n* = 100) Spinal group (*n* = 100)	Spinal anesthesia increased significantly the chance of IVF success (*p* < 0.001)	—	—	—

Milanini et al. 2008 [9]	Local anesthesia versus remifentanil	548 total Group I (*n* = 274) Group II (*n* = 274)	—	—	—	NS

Cerne et al. 2006 [4]	Preovarian block (POB) versus paracervical block (PCB)	183 total POB group (*n* = 96) PCB group (*n* = 87)	NS	NS	—	NS

Wilhelm et al. 2002 [6]	Monitored anesthesia care (MAC) with remifentanil versus GA	251 total GA group (*n* = 132) MAC group (*n* = 119)	MAC had a greater pregnancy rate (*p* < 0.05)	NS	NS	NS

Hammadeh et al. 1999 [8]	GA versus sedation	202 total Sedation group (*n* = 96) GA group (*n* = 106)	NS	NS	NS	The number of collected oocytes was significantly higher with general anesthesia (*p* < 0.0001)

Ng et al. 1999 [3]	Paracervical block with 1.5% lignocaine (group A) versus normal saline (group B) versus no local injection (group C)	135 total Group A (*n* = 45) Group B (*n* = 45) Group C (*n* = 45)	NS	—	—	NS

Christiaens et al. 1998 [7]	Propofol versus paracervical local anaesthetic block (PCB)	202 total Propofol group (*n* = 101) PCB group (*n* = 101)	NS	NS	NS	—

Botta et al. 1995 [5]	Epidural anesthesia (group A) versus Sedation (group B)	148 total Group A (*n* = 44) Group B (*n* = 104)	NS	NS	NS	—

NS: no significant difference, —: not under investigation.

**Table 2:** Details of trials investigating general anesthesia's (GA's) potential toxicity.

First author/year	GA versus other techniques	Sample size	Pregnancy rates	Fertilization rate	Oocytes
Aghaamoo et al. 2014 [2]	GA versus spinal analgesia	164 total GA group (*n* = 83) Spinal group (*n* = 81)	Practicing spinal anesthesia is significantly related to increased chance of chemical pregnancy (*p* = 0.043)	—	—

Azmude et al. 2013 [1]	GA versus spinal anesthesia	200 total GA group (*n* = 100) Spinal group (*n* = 100)	Spinal anesthesia increased significantly the chance of IVF success (*p* < 0.001)	—	—

Wilhelm et al. 2002 [6]	GA versus monitored anesthesia care (MAC) with remifentanil	251 total GA group (*n* = 132) MAC group (*n* = 119)	MAC had a greater pregnancy rate (*p* < 0.05)	NS	NS

Hammadeh et al. 1999 [8]	GA versus sedation	202 total GA group (*n* = 106) Sedation group (*n* = 96)	NS	NS	The number of collected oocytes was significantly higher with GA (*p* < 0.0001)

Christiaens et al. 1998 [7]	GA versus paracervical local anesthetic block (PCB)	202 total GA group (*n* = 101) PCB group (*n* = 101)	NS	NS	NS

NS: no significant difference, —: not under investigation.
